# A Popular Treatise on the Teeth

**Published:** 1848-04

**Authors:** 


*A Popular Treatise on the Teeth;* containing a history of the Dental Art, with anatomical descriptions of the Mouth and its appendages, and accounts of Chemical and Physiological Experiments on the Teeth. Also a full and accurate history of Ether or Letheon, for the Prevention of Pain, with directions for use. Designed for the use of families, and as a manual for the student and practical dentist. By MAYO G. SMITH, *Dental Surgeon. Illustrated by numerous engravings;* pp. 423, 12mo. Boston: JOHN P. JEWETT & Co.

The above is the title of a work which contains some very excellent advice. It is unique in its arrangement, and embodies considerable information, which, no doubt, would be valuable to the non-professional reader; but we fear it is too large for this class of individuals, and is too deficient in detail for the dental student or practitioner. It is difficult to write for the popular and professional reader at the same time, on a subject like dental surgery, without saying more than will be likely to be read by the one, or not enough to be serviceable to the other; and hence we doubt the propriety of attempting to embody in one work, information intended for the benefit and profit of two opposite classes of readers.

We had designed entering into a critical analysis of Mr. Smith's work, but delayed our intention until so late a period, that we have only time merely to announce its publication. We may, however, do so in a future number of the Journal.

---

